# Beyond Self-Blame: A Qualitative Study of the Changing Experience of Parental Guilt Following the Death of a Child

**DOI:** 10.3390/bs16101771

**Published:** 2026-09-28

**Authors:** Lucia Pelacho-Rios, Yolanda Ruiz-Ordoñez, Alejandro Sanchis-Sanchis, Gloria Bernabe-Valero

**Affiliations:** Facultad de Psicología, Universidad Católica de Valencia San Vicente Mártir, 46001 Valencia, Spain; lucia.pelacho@ucv.es (L.P.-R.); alejandro.sanchis@ucv.es (A.S.-S.); gloria.bernabe@ucv.es (G.B.-V.)

**Keywords:** parental bereavement, parental guilt, child loss, qualitative research, thematic analysis, grief adaptation, meaning reconstruction, prolonged grief

## Abstract

Parental guilt is one of the most pervasive emotional responses following the death of a child and has been consistently associated with adverse bereavement outcomes. However, little is known about how bereaved parents experience and integrate guilt over time. This qualitative study explored the lived experience of parental guilt, its relationship with the cause of death, and the processes that facilitate its integration. Semi-structured, in-depth interviews were conducted with 27 bereaved mothers and fathers and analysed using reflexive thematic analysis. Four themes were identified. First, the intensity and meaning of guilt differed according to the cause of death: sudden or potentially preventable deaths were associated with overwhelming self-blame, whereas anticipated deaths following illness elicited guilt related to caregiving and perceived inadequacy. Second, guilt was experienced as a multidimensional process, including protective, caregiving, counterfactual, survivor, and parental identity guilt. Third, participants retrospectively described changes in their relationship with guilt, from pervasive self-blame towards greater integration rather than complete resolution. Finally, meaning reconstruction, peer support, professional psychological support, and self-compassion facilitated this process. These findings highlight parental guilt as a dynamic process shaped by the circumstances of the loss, with implications for tailored bereavement assessment and intervention.

## 1. Introduction

Grief is a universal experience inherent to the human condition, arising after the loss of a significant other. It is conceptualised as a dynamic, continuous, and non-linear process characterised by the persistence of the bond with the deceased (Arnold et al., 2005).

The death of a child may challenge core aspects of personal identity and the parental caregiving role, as highlighted in previous research (Price & Jones, 2015). Recent contributions suggest that bereaved parents may experience a crisis in both personal and parental identity and may need to re-evaluate who they are after the loss. Y. Kim and Lee (2021) identified three identity-related patterns: reintegration, in which the death of the child is acknowledged and the parental role becomes oriented toward new goals; disintegration, in which the parent feels that they no longer have the right to live following the child’s death; and coexistence, characterised by oscillation between reintegration and disintegration.

In response to the loss, some studies have described parents’ efforts to reconstruct their identity. For instance, parents who had experienced an identity crisis reported great-er appreciation for life, attached less importance to material possessions, and placed greater value on spirituality (Markelin et al., 2025). Some parents also reported developing new forms of connection with their deceased child, such as engaging in voluntary activities with bereavement-related organisations, and described experiences of self-compassion, empathy toward others, and gratitude for life (Mohamed Hussin et al., 2025).

In relation to identity, meaning reconstruction is also a relevant area for understanding bereavement (Guillem Porta et al., 2013), as parents may feel that their assumptions about life and the world have been challenged following the loss. From this perspective, higher levels of meaning reconstruction have been associated with better bereavement outcomes during the first years after the loss (Neimeyer et al., 2006). Constructing a per-sonal narrative may help situate the loss within one’s life story and personal framework of meaning (Neimeyer et al., 2010), thereby supporting the redefinition of identity, interpersonal relationships, and one’s worldview. The literature has indicated that the presence of other children may support meaning reconstruction (Price & Jones, 2015), as may spiritual support and positive coping styles (Vig et al., 2021). Meaning reconstruction has also been associated with helping behaviours directed toward other parents who have experienced similar losses (Stevenson et al., 2017). Parents who experience greater difficulties finding meaning in the loss or identifying loss-related benefits have reported higher levels of both normative grief and maladaptive grief (Lichtenthal et al., 2010).

Several forms of meaning reconstruction have been described in parental bereavement. Meaning-making appears to be the most prominent, often involving biomedical explanations and, in some cases, counterfactual thinking. Other forms include identity re-construction, the maintenance of continuing bonds with the deceased, benefit-finding, and personal growth (Meert et al., 2015).

The specific circumstances of a death have been associated with the meanings at-tributed to the loss, perceptions of responsibility, and perceived preventability. Suicide bereavement represents a particularly complex form of loss. In this context, Baños-Chaparro (2022) noted that suicide bereavement has distinctive characteristics relative to other forms of bereavement, both in terms of its psychological and mental health consequences and bereaved relatives’ need to find an explanation for the loss. The sudden and violent nature of suicide has been associated with a greater need for explanation, as well as with feelings of guilt and prolonged distress.

However, the relationship between loss circumstances and the intensity or persistence of grief is not uniform. Some studies suggest that time since the suicide and the type of relationship with the deceased are not associated with grief or depression (Levi-Belz, 2023). Others indicate that grief may persist regardless of the time elapsed since the loss, the deceased child’s age, or the cause of death (Arnold & Gemma, 2008). In addition, difficulties in meaning reconstruction and unexpected or traumatic deaths have been associated with greater difficulties in adapting to bereavement (Goldstein et al., 2018; Keesee et al., 2008; Lichtenthal et al., 2010; Neimeyer et al., 2010).

Guilt is a relevant emotional experience in bereavement. It may be particularly salient when individuals perceive shortcomings in the care provided to the deceased person or experience unresolved conflicts (Albuquerque et al., 2025). Some studies have identified pain, yearning, and guilt as central dimensions of grief. In this regard, guilt has been closely associated with grief-related pain, yearning for the deceased person, and a perceived lack of meaning in life. Yearning has also been associated with feelings of guilt, although the direction of these associations is not univocal (Xiang & Li, 2026). In addition, prolonged grief has been associated with higher levels of guilt (Hofmann et al., 2026).

Beyond its associations with other components of grief, the scientific literature has described forms of guilt that may be relatively more adaptive or maladaptive. This dis-tinction should be understood as a useful conceptual framework rather than as a rigid, mutually exclusive clinical classification. Adaptive guilt has been linked to prosocial and reparative behaviours, whereas maladaptive guilt has been associated with poorer mental health and a diminished sense of identity (Joa & Newberg, 2021; Tilghman-Osborne et al., 2010). In its more adaptive form, guilt has been associated with empathy and sensitivity toward others; in its more maladaptive form, it has been associated with mental health problems, self-critical and self-destructive thoughts, and intrusive imagery that may hinder adaptation (Albuquerque et al., 2025; Anet et al., 2026). This distinction may be considered in relation to features such as the flexibility, intensity, content, and interference of guilt with adaptation. In this regard, some studies distinguish responsibility guilt, which has been associated with prolonged grief disorder, from self-blame, which has been associated with higher levels of grief (Joa & Newberg, 2021). Self-blame involves attributing responsibility for the death to oneself and regretting one’s decisions or actions. In some longitudinal studies, self-blame has been found to be more pronounced in the early period following the loss and to decrease over time (Stroebe et al., 2014). Self-blame has also been associated with greater difficulties in meaning reconstruction following the loss (M. A. Kim et al., 2024).

The cognitive model of guilt proposes that, in traumatic contexts, individuals may interpret the event in maladaptive ways, which may be associated with greater guilt (Pitcho, 2025). In this context, guilt may be particularly salient following violent or unexpected losses (Wagner et al., 2021). Metacognitive beliefs have also been described whereby relinquishing guilt may be perceived as equivalent to forgetting the deceased person, or guilt may be interpreted as punishment for remaining alive (Murray et al., 2021). Consistent with the above, higher levels of guilt have been associated with symptoms of complicated grief and with long-term adaptation difficulties (Li et al., 2019).

In this regard, some studies have examined the potential mediating role of guilt, although findings have not been consistent (Levi-Belz, 2023). With respect to prolonged grief, the empirical evidence is heterogeneous. Some studies suggest an association between guilt and prolonged grief (Serjouie et al., 2025). Studies of suicide bereavement have reported associations among guilt, time since the loss, and depressive symptoms (Wagner et al., 2021). Similarly, other research has indicated that guilt experienced between four and seven months after a loss is associated with higher levels of grief and depression, slower recovery from grief, and more persistent guilt-related cognitions (Stroebe & Schut, 2015). Guilt has also been positively associated with unfinished business, grief, rumination, and post-traumatic stress symptoms, and rumination has been proposed as a potential meditator of the association between guilt and prolonged grief (Albuquerque et al., 2025).

In the specific context of parental bereavement, guilt has been described as one of the negative emotions that may persist over time (Arnold & Gemma, 2008). Guilt may be more intense in complicated grief, and bereaved mothers and fathers may feel that they did not do enough for the deceased child, perceive themselves as responsible because they believe that they could have prevented the death, or experience survivor guilt (Li et al., 2019). Similarly, greater internalisation of guilt has been observed when bereaved parents perceive themselves as responsible for failing to protect their child or prevent the death and report difficulties in constructing meaning from the loss or maintaining an adaptive symbolic bond with their child. Higher levels of guilt have also been reported following accidental deaths (Peiró Ballestín et al., 2007). In cases of suicide, guilt may be directed toward one-self, the deceased child, or third parties (Back et al., 2026).

However, these manifestations are not uniform and do not occur in all loss contexts. The circumstances of the death and perceived responsibility may shape how guilt is experienced. Guilt may take different forms that are not necessarily mutually exclusive but may overlap within the same bereavement experience. Qualitative studies, particularly in the context of suicide bereavement, have described three forms of personal guilt: omission guilt, in which the bereaved person believes that they were unable to prevent the death; commission guilt, in which they attribute some influence on the death to their own actions; and survivor guilt, which is related to remaining alive after the death (Anet et al., 2026). Some findings have linked guilt to intrapersonal harm (Zeelenberg & Breugelmans, 2008). In addition, religious guilt has been reported among some bereaved parents who interpret the death of their child as punishment from a higher power (Vig et al., 2021). Some studies have also indicated that, following suicide loss, guilt directed toward third parties is associated with less time elapsed since the loss (Entilli et al., 2021).

In relation to these appraisals of responsibility, guilt in parental bereavement may arise primarily when parents believe that they did not care for or protect their child as they should have. Such experiences have been associated with the renegotiation of social roles and the search for meaning within a revised parental identity. In this regard, some parents have described a strengthening of spirituality and the maintenance of their parental role through their relationship with their other children (Mohamed Hussin et al., 2025).

Among these manifestations, survivor guilt may also be present in parental bereavement. From a cognitive perspective, this form of guilt is related to the belief that one does not deserve to remain alive following the death of a close person. A distinction has been made between content-related guilt, in which individuals believe that they did or failed to do something important, and existential guilt, which is related to being alive despite having no responsibility for the death (Murray et al., 2021).

Some studies have also described retrospective guilt related to the period of caring for the child. Higher levels of such guilt have been reported among parents who regret decisions they made or experience unresolved issues, such as not having discussed death, not having said goodbye, not having shared experiences that their child wished to have, or not having expressed affection toward their child. These experiences have also been associated with more severe symptoms of prolonged grief (Lichtenthal et al., 2020). This form of guilt, related to perceived failure to protect or care adequately for one’s child, has been identified as an element of relational identity, one of the dimensions most affected by the death of a child (Y. Kim & Lee, 2021). Taken together, these findings highlight the relevance of guilt in bereavement; however, research on guilt in the specific context of parental bereavement remains limited. Further research is needed to understand how parents make meaning of guilt following the death of a child and how guilt relates to prolonged grief disorder (Joa & Newberg, 2021).

Alongside guilt, shame may also play a relevant role in bereavement adaptation and has been associated with higher levels of post-traumatic stress (Shi et al., 2021; Yu et al., 2026). The literature has also identified shame among some parents whose children died by suicide (Duncan & Cacciatore, 2015). In general, within individualistic cultures, shame tends to be considered maladaptive and has been associated with negative self-evaluation, avoidance behaviours, and feelings of incompetence (Shi et al., 2021; Singh & Bhushan, 2025). Unlike guilt, which is typically linked to negative evaluations of specific actions or omissions, shame involves a more global negative evaluation of the self (Yu et al., 2026).

In this context, rumination is a relevant cognitive process in relation to guilt (Albuquerque et al., 2025). The death of a child has been associated with higher levels of grief rumination, and rumination has been linked to complicated grief, anxiety, and depression (Eisma et al., 2014). Grief rumination may include repetitive thoughts about one’s emotions, counterfactual thoughts about whether the death could have been prevented, repetitive thoughts about the perceived injustice of the death, rumination about others’ behaviour in response to the loss, and recurrent thoughts about the personal meaning of the loss (Eisma et al., 2014). In addition, rumination focused on unanswered questions, counterfactual thinking, and intrusive memories has been associated with shame and guilt (Murray et al., 2021). In suicide bereavement, higher levels of guilt have been reported alongside greater rumination and more frequent counterfactual thinking focused on what might have been done to prevent the death (Camacho et al., 2018).

Within the range of ruminative content, counterfactual thinking warrants particular attention. It refers to the construction of imagined alternatives to events that have already occurred and is frequently expressed through thoughts such as “if only…”. In bereavement, counterfactual thoughts may focus on what the bereaved person, the deceased person, or third parties did or failed to do, as well as on the circumstances of the death. Four relevant areas of counterfactual rumination may be distinguished: thoughts about what the deceased person did or did not do; thoughts about third parties directly or indirectly involved in the loss; perceptions of injustice regarding the contextual circumstances in which the death occurred; and self-focused ruminative thoughts concerning what the bereaved person could have done, or failed to do, to prevent the death. When such thoughts become persistent and repetitive, they may be associated with complicated grief, the chronicity of distress, and greater difficulty in meaning reconstruction (Neimeyer et al., 2019).

Overall, the available evidence indicates that guilt is a central emotion in parental bereavement. However, important gaps remain regarding how mothers and fathers experience guilt, how they retrospectively describe changes in their relationship with guilt throughout bereavement, and which factors shape its expression and integration. Most studies have examined guilt as a predictor or correlate of prolonged grief, whereas qualitative evidence capturing its meaning from bereaved parents’ own perspectives remains limited. Accordingly, the present study aims to explore mothers’ and fathers’ experiences of guilt following the death of a child, examining its relationship with the circumstances of the death, the different forms it may take, the changes that parents retrospectively describe over the course of bereavement, and the resources they identify as helpful for its integration.

## 2. Materials and Methods

### 2.1. Qualitative Design

Given the complexity, subjectivity, and deeply personal nature of guilt following the death of a child, a qualitative research design was considered the most appropriate approach for addressing the study aims. Rather than seeking to quantify the prevalence or intensity of guilt, the study aimed to understand how parents experienced, interpreted, and reconstructed these feelings throughout the bereavement process. A qualitative approach was therefore particularly well suited to capturing the complex, contextual, and evolving nature of these experiences.

The study was underpinned by a pragmatic epistemological orientation and employed reflexive thematic analysis (Braun & Clarke, 2022), an approach that is compatible with applied qualitative research while offering the interpretative flexibility needed to explore participants’ accounts in depth. Consistent with Braun and Clarke (2022)’s recommendations, the analytic approach was selected to ensure coherence between the study’s epistemological position, research aims, and analytic procedures.

Data were collected through semi-structured, in-depth interviews, which provided a flexible framework for exploring bereaved parents’ lived experiences while ensuring that key topics relevant to the research objectives were addressed consistently across participants. This approach enabled participants to describe their emotions, thoughts, and meaning-making processes in their own words, allowing the researchers to explore aspects of guilt that may not be anticipated in advance or adequately captured through more structured methods. Qualitative interviewing is particularly appropriate for investigating sensitive topics such as parental bereavement because it facilitates the development of trust, encourages participants to narrate emotionally significant experiences at their own pace, and allows interviewers to respond flexibly to issues that emerge during the conversation (O’Brien et al., 2014; Tong et al., 2007).

### 2.2. Participants

Participants were recruited using purposive sampling to identify mothers and fathers who had experienced the death of a child and were willing to participate in an in-depth interview about their bereavement experiences. Initial participants were recruited through three main pathways within the province of Valencia, Spain: Grupo Renacer, a bereavement support association with established links to the university where the study was conducted; the specialised Bereavement Unit of the university clinics; and personal contacts of members of the research team who met the study criteria and were informed about the possibility of participating. Subsequently, snowball sampling was employed, whereby participants shared information about the study with other bereaved parents who might be interested in participating. This recruitment strategy facilitated access to individuals with direct experience of the phenomenon under investigation while respecting the sensitive nature of the topic.

Eligibility criteria included being a mother or father who had experienced the death of a child after birth, regardless of the child’s age at death or the time elapsed since the loss. Deaths occurring during the neonatal period, childhood, adolescence, or adulthood were all considered eligible. This broad inclusion criterion was adopted because the study sought to explore the evolution and meaning-making of guilt across the bereavement process rather than acute grief reactions. Participants were also required to be willing and able to participate in an in-depth interview and to provide written informed consent. Individuals whose loss occurred during pregnancy, those considered by the research team to be in an acute phase of bereavement, or those presenting severe mental health conditions or communication difficulties that could compromise their ability to participate were excluded. Eligibility was assessed during the initial contact by members of the research team, all trained psychologists with experience in bereavement research, to ensure that participation was ethically appropriate and that participants were emotionally prepared to take part in the interview.

The sample was guided by the principle of information power, whereby participants’ direct experience of parental bereavement was expected to provide rich and relevant in-formation to address the study aims (Malterud et al., 2016). Consistent with this principle, sample size was determined by the specificity of the research question, the relevance of participants’ lived experience, and the richness of the interview data rather than by the achievement of data saturation. A total of 27 bereaved parents participated in the study, including 22 women and 5 men, with a mean age of 58.6 years (range = 37–83). The children were aged between 10 months and 44 years at the time of death (M = 21.0 years). Time since the child’s death ranged from 6 months to 60 years (M = 5.7 years), reflecting the study’s intention to capture experiences across different stages of the bereavement process. The causes of death reflected a broad range of circumstances, including illness, traffic accidents, suicide, drowning, and other accidental causes. Participants’ demographic and bereavement-related characteristics are provided in the Supplementary Materials. The purposive sampling strategy sought to include parents with varied bereavement experiences in order to explore the range and contextual complexity of parental guilt. However, the sample was not designed to support formal comparisons across causes of death, child age groups, sudden versus anticipated losses, or time since loss.

### 2.3. Data Collection

Semi-structured, in-depth interviews were conducted with bereaved mothers and fathers. The interview guide was developed by the two principal investigators, both health psychologists with expertise in bereavement, existential psychology, and qualitative research. The broader research team comprised psychologists and university lecturers with complementary expertise in health psychology, bereavement, emotional regulation, positive psychology, social psychology, and qualitative methodology. This combination of clinical, research, and methodological expertise informed the design of the interview guide and supported a sensitive exploration of participants’ experiences.

Prior to data collection, interviewers received study-specific training in the use of the semi-structured interview guide and the procedures established for conducting interviews on this sensitive topic, including rapport building, emotional debriefing, and ethical considerations. The interview guide was pilot-tested with two bereaved mothers to evaluate the clarity, comprehensibility, relevance, sequencing, and flow of the interview questions. Based on participants’ feedback and the research team’s observations, minor refinements were made to improve the wording and order of several questions while preserving the overall structure and objectives of the interview.

Interviews were conducted by trained members of the research team, all of whom had previous experience in qualitative interviewing. The interviewers comprised three female and one male psychologist. Following recruitment, interviews were typically scheduled within approximately two weeks of the initial contact, allowing sufficient time to coordinate participants’ and interviewers’ availability while avoiding unnecessary delays between enrolment and participation.

Participants were offered the choice of being interviewed either face-to-face or online. All chose to participate in face-to-face interviews, which were conducted either in a private room at the university or in participants’ homes, according to their preference. Given the emotional sensitivity of parental bereavement, conducting the interviews in person was considered the most appropriate approach for fostering trust, facilitating open communication, and creating a supportive environment in which participants felt comfortable discussing their experiences.

Each interview began with an informal conversation to establish rapport before addressing the bereavement experience. Throughout the interview, participants were encouraged to narrate their experiences in their own words, while interviewers used open-ended, follow-up, and probing questions to clarify meanings, explore emerging issues, and facilitate deeper reflection whenever appropriate. At the conclusion of the interview, participants were invited to share any remaining thoughts or concerns and were offered an opportunity for emotional debriefing, helping to ensure that they concluded the interview feeling supported.

The semi-structured interview guide provided sufficient flexibility to explore issues that emerged naturally during the conversation while ensuring that all topics relevant to the study aims were consistently addressed across interviews. This approach was considered particularly appropriate for exploring highly sensitive experiences such as parental bereavement, particularly the experience of guilt, which is often deeply personal, emotionally complex, and difficult to disclose. The flexibility of semi-structured interviewing enabled interviewers to respond empathically to participants’ narratives while maintaining methodological consistency (Hollway & Jefferson, 1997; O’Brien et al., 2014; Oplatka, 2018).

Interviews lasted between 25 and 43 min (M = 33.1 min). All interviews were audio-recorded with participants’ written informed consent.

Although the interview guide included questions about guilt, interviewers did not introduce this topic at the outset. Instead, they first invited participants to describe how their thoughts and emotions had changed over time following their child’s death (“How have your thoughts and emotions changed over time following your child’s death?”). Interviewers were trained to explore participants’ narratives progressively through open-ended and probing questions, attending to emotional reactions, beliefs, and self-evaluative thoughts that could reflect experiences of guilt without imposing this interpretation. When participants spontaneously identified guilt, interviewers explored its meaning, manifestations, and evolution over time in greater depth. When participants described experiences that appeared consistent with guilt but did not explicitly use that term, interviewers tentatively introduced the concept as a hypothesis (e.g., asking whether these experiences could be understood as feelings of guilt), allowing participants to confirm, reject, or further qualify this interpretation. Only if guilt had not emerged through these earlier stages was the topic addressed directly, using a normalising and non-judgemental prompt (e.g., “Some parents who have experienced the death of a child have described feelings of guilt. Was this also your experience?”). This progressive interviewing strategy was intended to minimise defensiveness, facilitate the disclosure of a highly personal and potentially difficult emotion, and distinguish between spontaneously emerging experiences of guilt and those acknowledged following more explicit exploration.

### 2.4. Procedure

Potential participants identified through the recruitment process were contacted by a member of the research team and invited to participate in the study. Some participants were known to members of the research team through previous professional or community contacts, whereas others were recruited through support groups and had no prior relationship with the interviewers.

During the initial contact, participants were informed about the aims of the study, the procedures involved, and the ethical safeguards in place. They were also informed that the interviewers were psychologists conducting research on parental bereavement. Eligibility was assessed according to the predefined inclusion and exclusion criteria. Those who agreed to participate received an information sheet, had the opportunity to ask questions, and provided written informed consent prior to data collection.

Interviews were scheduled at a mutually convenient time and location according to participants’ preferences. Before the formal interview began, interviewers reviewed the study information, confirmed participants’ willingness to continue, and reminded them that participation was voluntary and that they could decline to answer any question or withdraw from the study at any time without consequence.

Following each interview, audio recordings were securely stored and transcribed verbatim by trained student members of the research group who provided technical support for the study. All transcripts were subsequently reviewed for accuracy against the original audio recordings by members of the research team, anonymised by removing identifying information, and assigned a unique study code to ensure confidentiality. The anonymised transcripts constituted the dataset used for the thematic analysis.

The study was approved by the Ethics Committee of the Catholic University of Valencia (approval number: UCV/2022–2023/024) and was conducted in accordance with the ethical principles of the Declaration of Helsinki.

### 2.5. Data Analysis

Data were analysed using reflexive thematic analysis (RTA), following the six-phase approach proposed by Braun and Clarke (2022). Consistent with the study’s exploratory aims, the analysis was primarily inductive, with coding and theme development guided by close engagement with participants’ accounts rather than by a pre-existing theoretical framework. In accordance with RTA principles, themes were understood not as entities inherent in the data awaiting discovery but as interpretative constructions actively developed by the researchers through sustained engagement with participants’ narratives.

The analysis was situated within a pragmatic epistemological orientation. From this perspective, participants’ accounts were treated as meaningful representations of their experiences, while recognising that access to those experiences is necessarily mediated through language, context, and the researchers’ interpretative engagement. Accordingly, the analysis sought to develop an applied understanding of how parental guilt is experienced, changes over time, and may become integrated within the bereavement process, rather than to identify a single objective representation of guilt. The resulting interpretations therefore reflect an interaction between participants’ accounts, the study aims, and the researchers’ theoretically and clinically informed engagement with the data.

The analytic process began with repeated reading of the interview transcripts to promote familiarisation with the dataset and sustained engagement with patterns of meaning relevant to the research question. Initial codes were generated across the dataset and organised using Microsoft Excel. Coding remained iterative and flexible throughout the analysis, with codes revisited, revised, combined, or differentiated as the researchers’ understanding of the data developed. Rather than functioning as a fixed coding framework, the Excel file served as an organisational tool supporting this evolving interpretative process. Codes and associated extracts were subsequently examined for broader patterns of shared meaning, from which candidate themes were actively developed. Themes were iteratively reviewed and refined in relation to the coded extracts, the dataset as a whole, and the study aims, with particular attention paid to the central organising concept underpinning each theme and the relationships among themes. During analysis, attention was paid to contextual variation in participants’ accounts; however, the aim was not to conduct formal subgroup comparisons or to attribute particular manifestations of guilt to specific bereavement contexts.

The resulting themes were not conceptualised as independent or equivalent categories but as distinct and interconnected dimensions of participants’ accounts of guilt. During the interpretative phase, relationships among these dimensions were examined to develop a higher-order conceptual model linking the circumstances surrounding the death, manifestations of guilt, perceived changes in participants’ relationship with guilt over time, and resources and processes associated with its integration. The conceptual model was therefore not treated as an entity emerging directly from the data or as the product of an additional analytic procedure; rather, it represents an interpretative synthesis constructed through the researchers’ engagement with participants’ accounts and the relationships developed among the themes.

Regular analytical meetings were held throughout the analysis. These meetings provided opportunities for members of the research team to discuss developing interpretations, consider alternative readings of the data, question assumptions, and further refine the thematic structure and conceptual model. Consistent with a reflexive approach, differences in interpretation were not treated as coding discrepancies requiring resolution or consensus but were used as resources for reflexive dialogue and deeper engagement with the data.

Reflexivity was maintained throughout the analytic process. The research team comprised psychologists and university lecturers with complementary expertise in bereavement, health psychology, emotional regulation, positive psychology, social psychology, and qualitative methodology. These disciplinary and professional perspectives informed the researchers’ engagement with the data and were understood as resources for interpretation rather than as sources of bias external to the analytic process. At the same time, the team remained reflexively attentive to how prior clinical and research experience could shape the questions asked of the data and the interpretations developed.

To support transparency and allow readers to evaluate the relationship between participants’ accounts and the interpretations developed, the findings are illustrated with verbatim quotations selected to represent the breadth and diversity of participants’ experiences. The reporting of this study followed the Consolidated Criteria for Reporting Qualitative Research (COREQ; Tong et al., 2007), with the completed checklist provided in Supplementary Material File S1.

## 3. Results

The thematic analysis generated four interconnected dimensions of parental guilt. Rather than representing independent themes operating at the same analytic level, these findings capture complementary aspects of parents’ accounts: the contextual conditions shaping guilt, its different manifestations, participants’ retrospective accounts of changes in their relationship with guilt, and the resources and processes they associated with its integration.

### 3.1. Theme 1. The Weight of Guilt: How the Cause of Death Matters

Guilt emerged as one of the most pervasive emotional responses following the death of a child. However, participants’ narratives revealed that its intensity and meaning varied according to the circumstances surrounding the child’s death. While most parents questioned themselves to some extent, those whose children died suddenly and under circumstances perceived as potentially preventable described guilt as immediate and overwhelming. In contrast, parents whose children died following prolonged illnesses also experienced guilt, but this emotion was more often associated with caregiving decisions, helplessness, and the recognition that the illness had progressed beyond their control. These observations are presented as contextual variations within individual accounts rather than as formal comparisons between bereavement groups.

Among parents who experienced sudden and unexpected losses, guilt was characterised by persistent counterfactual thinking. Participants repeatedly revisited the events leading up to the death, mentally reconstructing alternative actions that might have changed the outcome. One mother whose daughter died from heart disease reflected this ongoing search for alternative explanations:

“There is always that feeling of ‘What if…?’ What if I had done something more? What if we had called the doctor earlier?”

(P12)

Similarly, a mother whose daughter died in a traffic accident described an almost obsessive tendency to mentally reconstruct what had happened, repeatedly returning to decisions she believed could have prevented the tragedy:

“If I hadn’t let her go… if I had brought her back with me… a thousand different thoughts. I know none of that changes anything now, but the feeling of guilt is still there.”

(P26)

By contrast, parents whose children died following prolonged illnesses also experienced guilt, but this emotion was generally expressed differently. Rather than attributing direct responsibility for the death, they questioned whether they had done enough to protect, comfort, or adequately care for their child throughout the illness. Their narratives reflected an ongoing search for possible omissions or missed opportunities despite recognising the inevitability of the disease. As one mother stated:

“Of course I felt guilty. As parents, we want to protect our children from everything. I even found myself wondering whether something in our home had caused his illness… I questioned everything.”

(P19)

Nevertheless, guilt was not universal. A small number of parents clearly differentiated between the pain associated with losing a child and personal responsibility for the death itself. These participants acknowledged reflecting on their actions but ultimately rejected self-blame, emphasising that the circumstances had been beyond their control. As one mother explained:

“I honestly don’t feel guilty. I couldn’t have prevented the accident. I gave her everything I could throughout her life, and in that sense, I’m at peace.”

(P13)

Overall, the findings suggest that although guilt was a common feature of parental bereavement, its expression depended not only on its intensity but also on how parents interpreted the circumstances surrounding their child’s death. Rather than representing a uniform emotional response, guilt took qualitatively different forms according to the cause of death. These distinct manifestations are explored in the following theme.

### 3.2. Theme 2. Different Manifestations of Guilt: A Multidimensional Experience

Although participants frequently referred to guilt as a single emotional response, closer examination of their narratives revealed multiple, interconnected foci of self-blame. These were not mutually exclusive categories; rather, different manifestations could co-occur within the same account and reflect different aspects of how parents interpreted responsibility following their child’s death. Five recurrent manifestations were identified: protective guilt, caregiving guilt, counterfactual guilt, survivor guilt, and parental identity guilt.

These manifestations were not mutually exclusive but reflected different foci of guilt within parents’ accounts. Protective guilt centred on the perceived failure to keep the child safe; caregiving guilt focused on perceived inadequacy in caring for the child or alleviating their suffering; counterfactual guilt involved repetitive thoughts about alternative actions or outcomes; survivor guilt concerned remaining alive or experiencing positive emotions after the child’s death; and parental identity guilt involved broader evaluations of one’s adequacy as a parent. Thus, a single account could contain more than one manifestation, with the same experience potentially involving, for example, a perceived failure to protect expressed through repeated counterfactual thoughts and subsequently interpreted as evidence of being an inadequate parent.

#### 3.2.1. Protective Guilt

Protective guilt centred on the perceived failure to keep the child safe. It was particularly evident following sudden and unexpected deaths, when parents focused on specific actions or omissions that, in retrospect, they believed might have prevented the loss. Rather than questioning their love for their child, they questioned whether they had fulfilled their fundamental responsibility to protect them. One father whose daughter drowned described this experience:

“I felt overwhelming guilt. I didn’t see her, I didn’t hear her, even though I was right there beside her… At the beginning it was unbearable… I still feel guilty. I haven’t been able to get rid of it completely.”

(P17)

Across participants, this perceived failure to protect reflected a disruption of one of the most fundamental aspects of parental identity. Parents often described an enduring belief that they should somehow have been able to keep their child safe, focusing on specific actions or omissions that, in retrospect, became associated with personal responsibility. As one mother explained:

“I felt guilty for not paying enough attention to her or looking after her more carefully. In fact, the poisoning she suffered was caused by something I had bought for her.”

(P14)

#### 3.2.2. Caregiving Guilt

In contrast, caregiving guilt did not focus on the child’s death itself but on the care provided before death, particularly among parents whose children died following prolonged illnesses. Rather than questioning whether they could have prevented the death, these parents questioned whether they had made the right medical decisions, recognised symptoms early enough, alleviated their child’s suffering, or done everything possible to care for and accompany them throughout the illness. This form of guilt was characterised by feelings of helplessness in the face of their child’s suffering, even when parents recognised that the course of the disease had been beyond their control. As one mother explained:

“In that sense, I feel at peace because I know there was nothing more we could have done to save her. But sometimes I did feel guilty because when she struggled to breathe and I saw her suffering, I couldn’t do anything. I felt powerless, as if I wasn’t protecting my daughter.”

(P12)

For some parents, guilt also extended to communication with their child during the final stages of illness, particularly concerns about whether they had said or done enough to provide reassurance and emotional support. One mother described repeatedly speaking to her son during his final moments, wanting to ensure that he knew his family was present:

“I kept talking to him because I wanted him to know that we were all there with him, that we hadn’t abandoned him for a single minute. I kept thinking what a terrible remorse I would have felt if I had left and my son had died without me there.”

(P10)

#### 3.2.3. Counterfactual Guilt

Several parents described an enduring preoccupation with what they should have done differently. In this study, counterfactual guilt referred to guilt expressed through repetitive counterfactual thinking, particularly thoughts about alternative actions or outcomes that might have changed the course of events. Unlike protective guilt, which focused on a perceived failure to protect their child, this manifestation revolved around endless hypothetical alternatives that emerged only in retrospect. Participants repeatedly described becoming trapped in “what if” thinking, mentally replaying past decisions and ordinary moments while recognising that these reflections could no longer alter the outcome. As one participant reflected:

“I kept thinking, ‘What if I hadn’t left? What if I had stayed? I should have done this… I should have said that…’ With therapy I realised I couldn’t have done any more, but at the beginning those thoughts were constant.”

(P09)

Similarly, another mother described repeatedly reconstructing seemingly insignificant decisions, wondering whether one of them might somehow have contributed to her son’s death:

“For more than two years, I kept wondering whether taking him to the dermatologist had somehow contributed to what happened. I couldn’t stop thinking that if I hadn’t insisted on that treatment, maybe none of this would have happened.”

(P05)

Other participants summarised this pattern of thinking more broadly, describing how their minds became occupied by endless alternative scenarios despite knowing that nothing could change the past:

“’What if we had gone somewhere else? What if we had changed our plans?’ Thinking about the past only destroys you because nothing will change what has already happened.”

(P02)

#### 3.2.4. Survivor Guilt

For several parents, guilt was unrelated to responsibility for the death itself and instead stemmed from remaining alive while their child had died. Participants described feeling guilty for experiencing moments of happiness, celebrating birthdays, enjoying meals, or simply continuing with daily life. The very act of living became emotionally conflictive. One mother explained:

“The first guilt is being alive while your child is not. I didn’t even want to celebrate my birthday because my daughter could no longer celebrate hers. I felt guilty for living.”

(P25)

Similarly, another mother described feeling guilty after engaging in ordinary activities that her daughter could no longer enjoy:

“How could I go out for dinner? How could I cut my hair? How could I eat something my daughter loved? Nobody sees the guilt you feel afterwards.”

(P26)

#### 3.2.5. Parental Identity Guilt

Parental identity guilt differed from the other manifestations in that the focus of evaluation extended beyond the circumstances of the death to parents’ broader sense of adequacy as mothers or fathers. Rather than focusing exclusively on whether they had caused the death or failed to prevent it, some parents questioned whether they had been good enough parents throughout their child’s life. This form of guilt could nevertheless overlap with protective or caregiving guilt when specific perceived failures were interpreted as evidence of being an inadequate parent. As one mother stated:

“My real guilt is asking myself whether I was a good enough mother… Did I do it well? That’s the question that stays with me.”

(P01)

Conversely, some participants explicitly rejected guilt because they believed they had fulfilled their parental role to the best of their abilities:

“I honestly don’t feel any guilt. There was nothing I could have done to prevent the accident. She was already a 26-year-old woman with her own life. She worked, travelled, and made her own decisions. Obviously, I couldn’t tie her to a chair to keep her safe.”

(P13)

“I don’t feel guilty because I gave my son everything I possibly could from the very beginning.”

(P10)

Taken together, these findings indicate that parental guilt cannot be understood as a single emotional experience. Instead, guilt emerged as a multidimensional process, with different manifestations reflecting parents’ attempts to make sense of their child’s death and to reconcile the enduring responsibilities, expectations, and identities associated with parenthood.

### 3.3. Theme 3. Changes in the Experience of Guilt: Participants’ Retrospective Accounts

Participants retrospectively described their relationship with guilt as changing over the course of bereavement rather than remaining static. Although the accounts varied in their timing and intensity, many parents recalled an early period in which guilt was experienced as particularly pervasive and emotionally overwhelming, followed by a gradual shift towards greater questioning, contextualisation, and integration. Importantly, these accounts represent participants’ retrospective perceptions of change rather than a longitudinally observed developmental trajectory. During the early stages of bereavement, guilt was described as pervasive and overwhelming, dominating parents’ thoughts and leaving little room for other emotions. Participants recalled becoming trapped in repetitive self-blaming thoughts, mentally reconstructing the circumstances surrounding the death and repeatedly asking themselves what they could have done differently. As one father explained:

“At the beginning it was unbearable… the guilt was overwhelming. I couldn’t stop thinking about what I should have done differently.”

(P17)

In their retrospective accounts, many parents described a gradual reduction in the intensity of these intrusive thoughts. Importantly, these accounts did not suggest that guilt necessarily disappeared. Instead, participants recalled that it became less constant and less emotionally consuming. Some parents explained that they continued to experience moments of self-blame, but these episodes became increasingly situational, often triggered by anniversaries, family events, or everyday situations that reminded them of their child. The same participant later reflected on this gradual transformation:

“I still feel guilty. I don’t think it will ever disappear completely. But now it doesn’t control my whole day like it used to.”

(P17)

Several parents also described a growing discrepancy between what they understood rationally and what they continued to experience emotionally. Although they recognised that they were not objectively responsible for their child’s death, emotional guilt often persisted independently of this cognitive understanding. One mother illustrated this tension:

“I know I couldn’t have prevented the accident… rationally I know that. But emotionally, the guilt still comes back.”

(P26)

For some participants, this process culminated in a gradual integration of guilt into their ongoing bond with their child. Rather than trying to eliminate guilt, they described accepting its presence while preventing it from defining their identity or daily functioning. In this sense, adaptation was not characterised by the absence of guilt but by a transformation in its meaning and influence. As one participant expressed:

“You keep blaming yourself until you eventually realise it wasn’t your fault. You can’t move forward until you let go of that guilt. We all feel guilty, but guilt doesn’t serve any purpose. It only hurts you. It’s a way of distracting yourself from the pain, of focusing your attention on something else.”

(P14)

Overall, participants’ narratives suggest that parental guilt follows a process of transformation rather than simple resolution. Instead of disappearing over time, guilt gradually changed its role within parents’ lives. Initially experienced as an overwhelming and pervasive emotion, it progressively became questioned through cognitive reappraisal while remaining emotionally present, eventually evolving into a more integrated experience that no longer dominated everyday functioning. Taken together, these retrospective accounts suggest a perceived transformation in the role and experience of guilt, although the timing and form of this change varied across participants. This interpretation is summarised in Figure 1.

### 3.4. Theme 4. Resources and Processes Associated with the Integrating of Guilt

Participants did not describe a single event or intervention that resolved their feelings of guilt. Instead, they retrospectively described adaptation as involving multiple interpersonal, cognitive, and emotional resources that they experienced as helpful in living with and reinterpreting guilt. Rather than describing the elimination of guilt, participants spoke of developing ways to reinterpret self-blame, recognise the limits of their responsibility, and relate to themselves with greater compassion. Four interconnected resources and processes emerged from the interviews: sharing the burden with others, reconstructing meaning, receiving professional support, and cultivating self-compassion and acceptance.

#### 3.4.1. Sharing the Burden with Others

For some participants, sharing their experiences with others was retrospectively described as an important source of relief and understanding. Support groups, particularly *Renacer*, a peer-support network for bereaved parents, provided a unique space where parents felt understood without needing to justify or explain their emotions. Sharing experiences with others who had endured a similar loss was described as helping to normalise feelings of guilt and reduce the sense of isolation that many had experienced. One participant explained:

“When I discovered Renacer, I realised that was where I needed to be. I didn’t have a husband I could talk to in the same way, and I didn’t want to place that burden on my children. There, everyone understood without me having to explain anything.”

(P25)

Rather than removing guilt, these shared experiences helped parents place their emotions into a broader context, allowing them to recognise that self-blame was a common response following the death of a child rather than evidence of personal failure.

#### 3.4.2. Reconstructing Meaning

A second process that participants retrospectively associated with living with guilt involved reconstructing the meaning of the loss. As time passed, many parents described moving away from repeatedly asking *“Why did this happen?”* towards asking *“How can I continue living while honouring my child’s memory?”* This shift did not erase guilt but fundamentally altered its significance, allowing parents to integrate the loss into their life story rather than remaining trapped in endless counterfactual thinking.

For some parents, this reconstruction involved developing a new personal or spiritual understanding of the loss, enabling them to interpret the experience within a broader framework of meaning. As one mother explained:

“I have given it a spiritual meaning. I believe it had to happen that way… It has made me see things differently that I had never seen before.”

(P21)

For others, meaning reconstruction emerged through the gradual acceptance that the past could not be changed. Rather than continuing to search for impossible explanations or alternative outcomes, they redirected their emotional energy towards preserving their child’s memory, maintaining an enduring bond, and rebuilding their own lives. As one mother reflected:

“There’s no point in getting stuck in a loop over something you can’t change. That moment has passed, and nothing you say or think will change it. We don’t have a time machine. We have to learn to live with that. Instead, we try to keep his memory and his life alive because we believe he is still with us.”

(P02)

In this way, guilt gradually became one aspect of grief rather than its defining feature.

#### 3.4.3. Receiving Professional Support

Several participants retrospectively described professional psychological support as helpful in reconsidering feelings of guilt and developing a more compassionate interpretation of their experiences. Participants did not portray therapy as a way of making guilt disappear but as a safe environment in which difficult emotions could be expressed openly and experienced without judgement. As one participant reflected:

“I liked being able to talk openly. To cry when I needed to, to feel understood by that person, and to receive help.”

(P21)

However, experiences of therapy were not universally positive. Some parents explained that professional support was only helpful when clinicians acknowledged the uniqueness of parental bereavement and avoided offering premature reassurance or simplistic explanations. These accounts suggest that interventions should focus not on eliminating guilt but on helping parents understand, contextualise, and gradually integrate it.

#### 3.4.4. Cultivating Self-Compassion and Acceptance

Some participants described developing greater self-compassion and acceptance as an important part of learning to live with guilt. Rather than continuing to judge them-selves according to unrealistic expectations of parental responsibility, they described gradually acknowledging that they had acted with the knowledge, resources, and circum-stances available to them at the time. This shift from self-criticism towards greater self-understanding was perceived by participants as an important aspect of adapting to the loss. One participant explained:

“Acceptance is the key. You realise that blaming yourself doesn’t help your child, and it only prolongs your own suffering.”

(P20)

Across interviews, acceptance was not described as forgetting the child or becoming free from guilt. Instead, parents portrayed it as learning to coexist with guilt without allowing it to define their identity or dominate everyday life. In this sense, adaptation was characterised not by the disappearance of guilt but by a transformation in the relationship parents maintained with it.

Overall, participants’ narratives suggest that adapting to parental guilt is less about eliminating self-blame than about transforming the way it is experienced. The findings indicate that the cause of death influences not only the emotional weight of guilt but also the forms it adopts, its evolution over time, and the processes that facilitate its integration. Together, these interconnected findings give rise to an emergent conceptual model of parental guilt following the death of a child, summarised in Figure 2. The model provides an interpretive synthesis of the relationships identified across participants’ accounts. The circumstances surrounding the child’s death shaped how guilt was experienced and interpreted, while guilt itself encompassed several interconnected and potentially overlapping manifestations. Participants also retrospectively described changes in their relationship with guilt and identified several interpersonal, cognitive, and therapeutic processes that they associated with greater integration.

## 4. Discussion

The present study aimed to qualitatively explore parents’ experiences of guilt following the death of a child. The findings indicate that guilt does not constitute a uniform emotional response but rather a multidimensional phenomenon that is shaped by the context of the loss and was retrospectively perceived by participants to change over the course of bereavement. The value of the present analysis lies not in proposing four discrete and equivalent categories but in showing how different dimensions of parental guilt are interconnected within parents’ narratives. The emergent conceptual model should therefore be understood as an interpretive synthesis of the thematic findings rather than as developmental, causal, or generalisable across bereavement groups.

Most participants reported experiencing guilt, although its intensity and expression varied according to the cause of death. In contrast, some parents described profound grief without feelings of guilt. These findings are consistent with previous research demonstrating that guilt is a prominent component of parental bereavement, although its intensity differs substantially depending on the circumstances surrounding the child’s death (Albuquerque et al., 2025; Camacho et al., 2020).

Parents whose children died suddenly and unexpectedly under circumstances perceived as potentially preventable described an immediate and overwhelming sense of guilt. This finding is consistent with evidence identifying guilt as one of the most prevalent and enduring emotional responses following sudden or violent loss (Bell et al., 2012; Tal et al., 2017). Their accounts were characterised by persistent counterfactual thinking, a pattern that is consistent with research identifying the nature of sudden deaths as a specific risk factor for the development of intense guilt (Camacho et al., 2018). It also aligns with studies suggesting that guilt is associated with rumination, expressed through counterfactual thinking, as a strategy for emotion regulation (Boelen et al., 2006; Van der Houwen et al., 2010). Sudden child death resulting from accidental, violent, or otherwise unexpected causes has been associated with higher levels of guilt than other types of child loss (Duncan & Cacciatore, 2015), as well as with increased rates of complicated grief, with part of this elevated risk appearing to be attributable to guilt, self-blame, or shame (Champion & Kilcullen, 2025).

Another important contribution of the present study was the identification of five interconnected manifestations of guilt (protective guilt, caregiver guilt, counterfactual guilt, survivor guilt, and parental identity guilt). These manifestations are not mutually exclusive and may overlap and co-occur within the same parent’s account, particularly protective counterfactual, and parental identity guilt; they are therefore better understood as interconnected foci of guilt than as discrete diagnostic categories. This finding supports previous research highlighting the conceptual ambiguity of treating guilt as a unitary emotional construct within the bereavement literature (Li et al., 2014). Beyond confirming the multidimensional nature of guilt, the present findings demonstrate that these dimensions are also evident within the specific context of parental bereavement. This multidimensional conceptualisation is further supported by the taxonomy of guilt subtypes recently identified in a thematic analysis of interviews with adults bereaved by suicide, which distinguished omission guilt, commission guilt, and survivor-related guilt (Anet et al., 2026). The convergence of structurally similar categories despite differences in samples, countries, and causes of death suggests that these dimensions are not culture-specific but may instead reflect relatively generalisable psychological processes underlying traumatic bereavement. Nevertheless, further research involving culturally diverse samples and comparative study designs is needed to establish more robustly the extent to which these processes can be generalised across sociocultural contexts and different types of loss.

Protective guilt emerged as the most frequently reported form of guilt and was particularly evident among parents whose children had died suddenly and unexpectedly, in agreement with previous studies (Duncan & Cacciatore, 2015; Lee et al., 2024; Shalev et al., 2023). Within parental bereavement, this form of guilt appears to be primarily related to perceived responsibility, retrospective beliefs about control, and moral standards that shape parental identity, rather than to objective causal responsibility (Duncan & Cacciatore, 2015; Lee et al., 2024).

In contrast, parents whose children died following a prolonged illness described a form of guilt that was less focused on the preventability of the outcome and more strongly related to questioning the adequacy of the care and protection they had provided. This pattern is consistent with previous research (Lichtenthal et al., 2020; Yi et al., 2023) and has been associated with greater psychological distress; rumination; regret; and, in some parents, prolonged or complicated grief (Lichtenthal et al., 2020; Serjouie et al., 2025; Zetumer et al., 2015).

Caregiver guilt was primarily characterised by persistent doubts about having failed to do enough or having fallen short in fulfilling one’s parental role (Gallego-Alberto et al., 2020; Lichtenthal et al., 2020; Mohamed Hussin & Mohd Sabri, 2023; Zetumer et al., 2015). This form of guilt appears to stem from highly demanding parental caregiving standards, conflicts between competing roles, and negative evaluations of one’s own caregiving performance (Gonyea et al., 2008; Mohamed Hussin & Mohd Sabri, 2023).

In contrast, parents whose children died suddenly described a qualitatively different form of guilt. Their narratives revealed a pronounced pattern of counterfactual thinking, consistent with the conceptualisation proposed by Eisma et al. (2014), who identified counterfactual thinking as one of the five core dimensions of grief-related rumination, together with rumination about meaning, relationships, injustice, and emotional reactions. This repetitive cognitive pattern may contribute to the maintenance of psychological distress by functioning as a short-term avoidance strategy. Rather than accepting the reality of the loss, bereaved parents repeatedly focus on its causes, consequences, and hypothetical alternative scenarios, thereby avoiding the painful reality of the death and the deeper emotional suffering associated with it. Although this pattern may temporary relieve emotional distress, participants’ accounts suggest that it may also be associated with the persistence of distress over time (Camacho et al., 2018). This pattern may help contextualise why participants described counterfactual guilt as particularly intrusive and difficult to regulate during the early stages of bereavement.

The present findings also provide a more nuanced understanding of counterfactual thinking in parental bereavement. Neimeyer et al. (2019) conceptualised “if only” thinking as a form of counterfactual cognition that may become repetitive and interfere with meaning reconstruction following bereavement, identifying the self, the deceased, relevant others, and circumstances of the death as possible foci. In the present study, counterfactual thinking was particularly closely tied to perceived parental responsibility. Parents repeatedly imagined alternative actions through which they might have prevented the death, reduced their child’s suffering, or acted differently. Importantly, these findings suggest that counterfactual thinking should not necessarily be understood as a discrete form of guilt that is independent of other manifestations. Rather, it may function as a cognitive process through which protective, caregiving, or parental identity guilt is expressed and maintained.

This distinction also suggests that rumination associated with caregiver guilt differs qualitatively from that underlying counterfactual guilt. In caregiver guilt, repetitive thoughts were not focused on constructing alternative scenarios in which the child’s death could have been prevented but rather on evaluating one’s own performance as a caregiver, a pattern that more closely resembles the “relationships” dimension of grief-related rumination described by Eisma et al. (2014). Although both forms of rumination have been associated with complicated grief, counterfactual thinking appears to show a substantially stronger relationship with adverse bereavement outcomes.

Another manifestation of guilt identified in the present study was survivor guilt. This form of guilt is characterised by feelings of distress related to remaining alive after the child’s death or by perceiving one’s own survival as unfair or undeserved (Bistas & Grewal, 2023; Murray et al., 2021), a pattern reflected in the narratives of several participants.

Unlike counterfactual guilt, which is typically linked to a specific action or omission, survivor guilt often involves a broader and more diffuse evaluation of one’s right to continue living or experiencing enjoyment. Phenomenologically, this pattern appears to be more closely related to shame than to guilt, according to the classical distinction between these two emotions. Whereas guilt involves the evaluation of a specific behaviour, shame reflects a global devaluation of the self (Singh & Bhushan, 2025). These findings suggest that future research on survivor guilt in parental bereavement should distinguish between its guilt- and shame-related components, as these dimensions may differ in their underlying mechanisms and responsiveness to therapeutic interventions.

Finally, some parents described what was classified as parental identity guilt. This form of guilt may represent a central component of parental bereavement and has been associated with more persistent grief reactions (Mohamed Hussin et al., 2025; Plews-Ogan et al., 2022; Zetumer et al., 2015). Parental identity guilt involves questioning whether one was a sufficiently good parent during the child’s lifetime and is closely related to research on identity reconstruction following the traumatic death of a child. A study of bereaved parents identified the reconciliation of social identity, the reconciliation of personal self-perceptions, and the search for meaning within a new parental identity as key psychological processes following traumatic child loss (Mohamed Hussin et al., 2025). These findings reinforce the notion that parental identity guilt should not be regarded as a consequence of counterfactual guilt but rather as a distinct dimension linked to the narrative reconstruction of the self. This interpretation is consistent with meaning reconstruction models, which identify identity reconstruction, meaning-making, benefit finding, and the maintenance of continuing bonds with the deceased as core processes of adaptation to bereavement (Meert et al., 2015).

In this context, it is important to note that not all parents experienced guilt. For some participants, their parental identity did not appear to have been fundamentally disrupted, as they believed they had done everything possible to fulfil their parental role. The absence of guilt may be more likely when parents are able to reintegrate their identity, redefine themselves beyond being “the parent of the deceased child,” or maintain their parental identity through memories of their child and continuing bonds with the deceased (Y. Kim & Lee, 2021; Payne et al., 2025).

The dynamic nature of guilt, as retrospectively perceived by participants, represents another key contribution of the present study. Rather than describing a linear disappearance of guilt, participants retrospectively described a gradual transformation from an intense and pervasive experience of self-blame to a less frequent, more contextualised, and progressively integrated emotional experience. Given the cross-sectional and retrospective design of the present study, these accounts should be understood as participants own retrospective perceptions of how their relationship with guilt had changed since the death of their child, rather than as evidence of an empirically established developmental trajectory. This perceived transformation is consistent with contemporary bereavement models, which conceptualise grief as a dynamic and oscillating process rather than a universal sequence of stages. The Dual Process Model proposes that bereaved individuals alternate between loss-oriented and restoration-oriented coping, such that adaptation requires moving back and forth between confronting the pain of the loss and attending to the demands of everyday life (Stroebe & Schut, 1999). The findings of the present study suggest that participants perceived guilt as following a similar oscillating pattern: although it may gradually lose its centrality, it can re-emerge in response to anniversaries, memories, or major life events. Such context-dependent reactivation should not necessarily be interpreted as evidence of therapeutic failure or chronic grief. Instead, these findings suggest that guilt should be evaluated in terms of its rigidity, intensity, and functional interference rather than its mere presence.

From a clinical perspective, the persistence of intense guilt may be particularly relevant. Quantitative studies have consistently reported associations between bereavement-related guilt, depression, and complicated or prolonged grief in both cross-sectional and longitudinal research (Li et al., 2019). Although these findings do not support the conclusion that guilt alone causes poor bereavement outcomes, they do suggest that guilt may function as a maintaining process and an indicator warranting more comprehensive clinical assessment. In particular, the coexistence of rigid self-blame, counterfactual rumination, avoidance, functional impairment, and hopelessness may signal a more complex grief response. Clinical assessment should therefore distinguish between painful but flexible guilt, which can coexist with moments of emotional connection and adaptive functioning, and a global, absolutist, and punitive form of guilt that becomes central to the parent’s identity. This distinctions is proposed as a potentially useful interpretative framework to guide clinical attention rather than as a validated diagnostic threshold; the cross-sectional, qualitative design of the present study does not allow thresholds between adaptive and pathological guilt to be empirically established.

This perceived transition from overwhelming guilt toward a more integrated experience may, from an interpretative standpoint, be understood as reflecting a process of cognitive reappraisal. According to Gross’s process model of emotion regulation (Gross, 1998), cognitive reappraisal involves reinterpreting the meaning of an emotionally activating situation in order to modify its emotional impact without suppressing the emotion or avoiding its processing. Several participants described guilt as initially providing a sense of control over events that could not otherwise be controlled and as a way of keeping the full emotional impact of the loss at a distance. From an interpretative perspective, these accounts may be understood as reflecting what can be conceptualised as an illusory sense of control and a form of experiential avoidance, an interpretation that extends beyond participants’ own explicit descriptions and should therefore be read as the authors’ theoretical reading of these accounts rather than as a directly demonstrated mechanism. Through what may be interpreted as cognitive reappraisal, however, many parents were, according to their own accounts, able not to eliminate guilt but to transform its regulatory function. Rather than perpetuating ruminative processes that maintained psychological distress, guilt gradually became integrated as a recognised—although not necessarily welcome—component of the bereavement experience. This interpretation is consistent with the findings of Camacho et al. (2018), who proposed that increasing levels of guilt are associated with greater difficulties in emotion regulation, underscoring the importance of intervening early in ruminative processes before they become chronic.

Parents in the present study described the resolution of guilt as a gradual process supported by multiple interconnected coping resources. One of the most important was sharing their emotional burden with other bereaved parents. Such exchanges appear to be particularly beneficial when they involve empathic, non-judgemental listening (Schoonover et al., 2022; Tognela et al., 2025). Support groups may provide a safe environment in which parents can openly express their emotions and talk about their child and the circumstances of the death without fear of being judged (Henning et al., 2021; Stevenson et al., 2017). They also help parents feel understood by others who have experienced a similar loss, thereby reducing feelings of isolation and of being fundamentally different from those around them (Berrozpe et al., 2024; Pelacho-Ríos et al., 2025).

Another resource associated with the integration of guilt, identified in the present study, was meaning reconstruction, consistent with Neimeyer’s model (Neimeyer, 2000a, 2000b), which proposes that adaptation to bereavement does not involve “getting over” the loss but rather integrating it into a coherent life narrative. The shift described by participants reflects this process of meaning reconstruction, which has been consistently associated with lower levels of complicated grief in the bereavement literature (Neimeyer, 2019). Participants reconstructed meaning in different ways, including through new personal or spiritual understandings of the loss. For many parents, spirituality provided a framework for making sense of the death, maintaining a continuing bond with the deceased child, re-evaluating personal values, and rebuilding a meaningful life. However, spirituality did not function uniformly across participants and, in some cases, intensified existential struggles when pre-existing beliefs no longer aligned with the reality of the loss (Mohamed Hussin & Aho, 2025; Neimeyer et al., 2021; Superdock et al., 2024).

Participants also described professional support as an important resource in the process of meaning reconstruction, although this experience was not universal and some reported less satisfactory encounters. Professional support appeared to be most beneficial when it did not seek to “resolve” or prematurely close the grieving process but instead provided a safe therapeutic relationship grounded in empathic presence and deep listening. Within this context, psychoeducation, existential and spiritual exploration, and emotion regulation strategies were perceived as particularly helpful in facilitating meaning reconstruction, maintaining continuing bonds with the deceased child, and reorganising life following the loss (Neimeyer et al., 2021; Vig et al., 2021).

Finally, self-compassion and acceptance emerged as two of the most important psychological resources for adaptation, as reflected in participants’ narratives and supported by the existing literature. Acceptance appears to function not as overcoming the child’s death but as integrating the loss into one’s identity, life story, and worldview. When this process occurs, it is typically accompanied by a transition from the initial disorganisation of grief toward a reconfigured life characterised by new values, renewed purpose, and new ways of maintaining a bond with the deceased child (Bogensperger & Lueger-Schuster, 2014; Neimeyer et al., 2010). Similarly, self-compassion appears to facilitate psychological adjustment by reducing self-criticism and guilt while promoting more adaptive emotion regulation through decreases in rumination, thereby supporting the reconstruction of meaning (Bogensperger & Lueger-Schuster, 2014; Zhang et al., 2022).

The findings of the present study have several direct implications for clinical practice with bereaved parents. First, the assessment of parental guilt should be sensitive to the cause of the child’s death. Sudden deaths or deaths perceived as preventable tend to elicit more immediate and counterfactual forms of self-blame, whereas deaths following prolonged illness are more commonly associated with guilt centred on caregiving and helplessness. Clinical assessments that fail to distinguish between these trajectories may result in generic interventions that do not adequately address parents’ lived experiences.

Second, the identification of five interconnected manifestations of guilt (protective guilt, caregiver guilt, counterfactual guilt, survivor guilt, and parental identity guilt) provides clinicians with a useful framework for case formulation by allowing interventions to target the specific content of guilt rather than treating it as a unitary construct. In particular, the distinction between survivor guilt and shame suggests that these experiences should be assessed and addressed separately, as they may involve different underlying mechanisms and require different therapeutic approaches.

Third, the dynamic and oscillating nature of guilt observed in the present study suggests that its re-emergence during anniversaries, memories, or significant life events should not automatically be interpreted as evidence of therapeutic failure or grief chronicity. Instead, such reactivation may represent an expected feature of the bereavement process. Consequently, clinical attention should focus on the rigidity, intensity, and functional interference associated with guilt rather than on its mere presence, with particular attention paid to parents whose self-blame becomes global, absolutist, and punitive, given its association with complicated or prolonged grief.

Finally, participants consistently reported that professional support was most helpful when it did not attempt to eliminate guilt or provide premature reassurance, but instead facilitated cognitive reappraisal, meaning reconstruction, and the development of self-compassion. These findings suggest that psychological interventions for parental bereavement should prioritise these therapeutic processes over approaches focused exclusively on symptom reduction. In addition, peer support groups may represent a valuable complement to individual psychotherapy by normalising feelings of guilt and reducing perceived social isolation.

A particular strength of this study is the inclusion of parents at markedly different stages of bereavement, including individuals whose loss had occurred decades earlier. This temporal breadth allowed the study to capture not only the immediate experience of guilt but also the ways in which its meaning and intensity may be reconstructed over the life course. In particular, the inclusion of older participants offered a unique opportunity to explore how parental guilt is understood from the perspective of later adulthood, providing insights that are rarely represented in the bereavement literature.

Second, the use of reflexive thematic analysis (Braun & Clarke, 2022), supported by regular analytical meetings, an evolving coding framework, and sustained reflexivity within a multidisciplinary research team, strengthens the interpretative rigour of the findings. Adherence to the COREQ reporting criteria further enhances methodological transparency and facilitates external appraisal of the study.

Nevertheless, several limitations should be acknowledged. First, the sample consisted predominantly of mothers, limiting the extent to which the findings can be generalised to bereaved fathers. Second, the cultural context of the sample should be considered when interpreting the results. Experiences of parental guilt are likely to be influenced by cultural, religious, and social factors shaping expectations regarding motherhood and fatherhood. Consequently, replication of the present conceptual model across diverse sociocultural contexts is warranted.

Building on the in-depth analysis of parental bereavement presented in this study, future research should adopt longitudinal designs that follow the same parents over time, allowing the developmental trajectory of guilt to be examined prospectively rather than inferred retrospectively. Future qualitative studies using purposive comparative sampling could also examine more precisely how parental guilt varies and evolves across different loss contexts. It would also be valuable to quantitatively develop and validate the conceptual model and the proposed taxonomy of five manifestations of guilt using psychometrically sound instruments capable of empirically distinguishing these dimensions and examining their differential associations with complicated and prolonged grief. Finally, replicating this study in samples with greater representation of bereaved fathers and across different cultural and religious contexts would help determine the generalisability of these findings and further advance the understanding of parental bereavement.

## 5. Conclusions

The present study demonstrates that guilt constitutes one of the central psychological processes in parental bereavement, while also representing one of its most complex dimensions. Rather than being a unitary emotional response, guilt emerged as a multidimensional experience whose expression and intensity were related to the circumstances surrounding the child’s death and to the ways in which parents interpreted their responsibility and reconstructed their identity following the loss. Participants also retrospectively described changes in their relationship with guilt, including a shift from intense self-blame towards greater integration, although the timing and nature of these changes varied across individuals.

The principal contribution of this study lies in the development of two complementary conceptual models that conceptualise parental guilt from a process-oriented perspective. The first summarises participants’ retrospective accounts of changes in their relationship with guilt, from intense initial self-blame towards greater integration, while the second illustrates the relationships among the cause of death, the different manifestations of guilt, and the resources and processes that participants associated with its integration. Together, these models extend current knowledge of parental bereavement by highlighting that adaptation may involve not the elimination of guilt but changes in how parents understand and live with it, including through meaning reconstruction, interpersonal support, professional psychological support, and self-compassion.

These findings contribute to shifting the understanding of guilt from a predominantly psychopathological perspective toward a more dynamic and integrative conceptualisation, providing a theoretical and clinical framework that may inform future research and the development of more individualised psychological interventions. Recognising the multiple manifestations of guilt and the changes that parents retrospectively describe in their relationship with it may help inform more tailored clinical care for bereaved parents, supporting approaches that seek not to eradicate guilt but to integrate it into a coherent life narrative that preserves the continuing bond with the deceased child while supporting the reconstruction of a meaningful life.

## Figures and Tables

**Figure 1:**
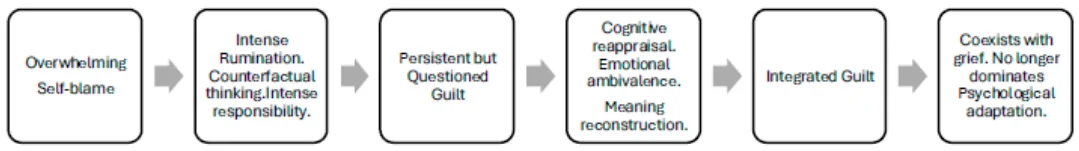
Emergent conceptual model illustrating participants’ retrospective accounts of changes in parental guilt following the death of a child.

**Figure 2:**
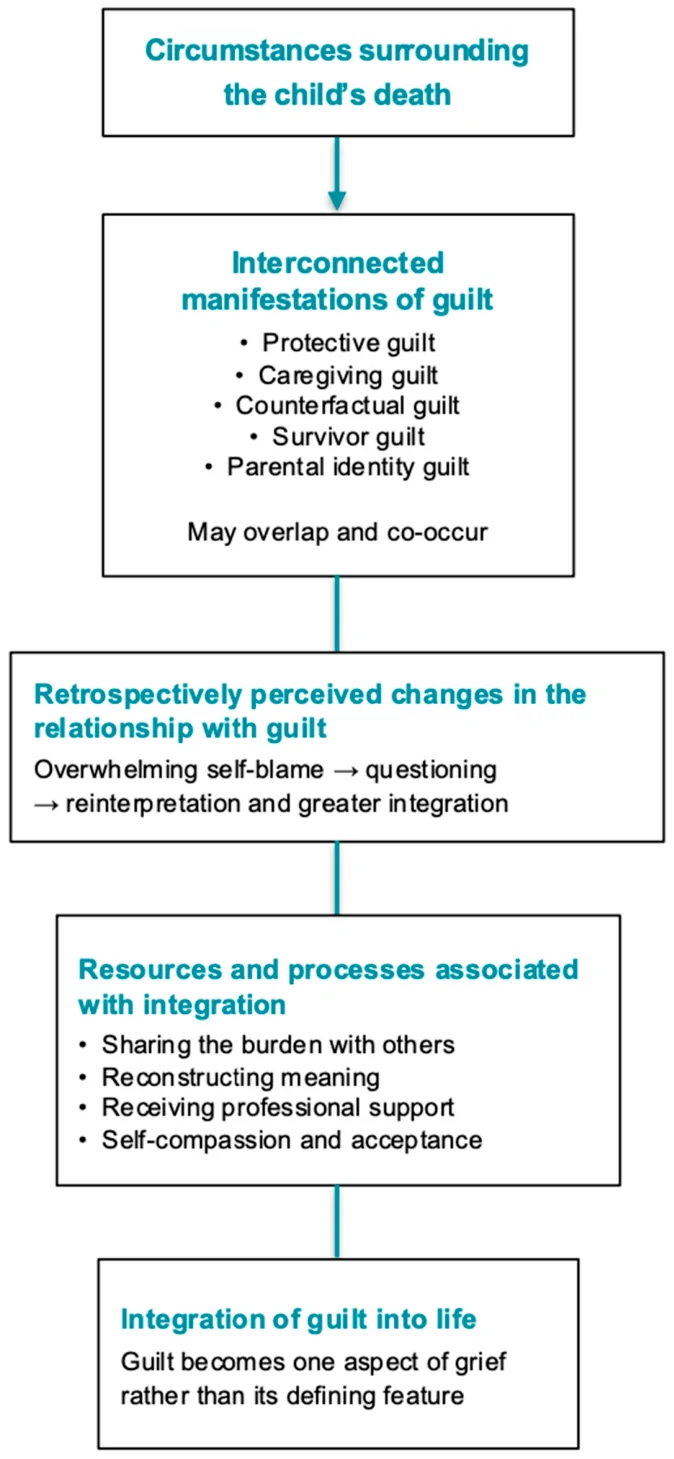
Interpretative conceptual model of parental guilt following the death of a child. **Note**. This model represents an interpretive synthesis of participants’ accounts. The identified manifestations of guilt may overlap and co-occur. Changes in the relationship with guilt reflect participants’ retrospective accounts rather than a longitudinally established trajectory. The re-sources and processes identified by participants as helpful should not be interpreted as causal pathways.

## Data Availability

The data presented in this study are available from the corresponding author on request due to ethical, private, and legal restrictions.

## Supplementary Materials

The following supporting information can be downloaded at: https://www.mdpi.com/article/10.3390/bs16101771/s1, Table S1. Participant Characteristics; Supplementary File S1. COREQ (Consolidated Criteria for Reporting Qualitative Research) Checklist.
