# Supplementary material for: Beyond Self-Blame: A Qualitative Study of the Changing Experience of Parental Guilt Following the Death of a Child
Source: Behav Sci (Basel). 2026 Sep 28;16(10):1771. doi: 10.3390/bs16101771 (PMC13701316; doi:10.3390/bs16101771)
Supplement: Supplementary file 1 [file behavsci-16-01771-s001.zip › File S1.pdf]

SUPPLEMENTARY MATERIALS S1

COREQ (Consolidated Criteria for Reporting Qualitative Research) Checklist

No.	Item	Guide questions/description	Reported on page No.
1	Interviewer/facilitator	Which author(s) conducted the interview or focus group?	7
2	Credentials	What were the researcher's credentials?	7
3	Occupation	What was their occupation at the time of the study?	7
4	Gender	Was the researcher male or female?	7
5	Experience and training	What experience or training did the researcher have?	7
6	Relationship established	Was a relationship established prior to study commencement?	6, 8
7	Participant knowledge of the interviewer	What did participants know about the researcher?	6
8	Interviewer characteristics	What characteristics were reported about the interviewer/facilitator (e.g., bias, assumptions, reasons, and interests in the research topic)?	7
9	Methodological	What methodological	6-7, 10

	orientation and theory	orientation was stated to underpin the study?	
10	Sampling	How were participants selected?	6
11	Method of approach	How were participants approached?	6
12	Sample size	How many participants were included?	6
13	Non-participation	How many people refused to participate or dropped out? Reasons?	N. R.
14	Setting of data collection	Where was the data collected?	7-8
15	Presence of non-participants	Was anyone else present besides the participants and researchers?	N.R.
16	Description of sample	What are the important characteristics of the sample?	Supplementary Table S1.
17	Interview guide	Were questions, prompts, or guides provided? Was it pilot-tested?	7-8
18	Repeat interviews	Were repeat interviews carried out?	N.A.
19	Audio/visual recording	Did the research use audio or visual recording?	8
20	Field notes	Were field notes made during and/or after the interviews?	N.R.
21	Duration	What was the duration of the interviews?	8
22	Data saturation	Was data saturation discussed?	6. Saturation was not used; sample

			size was guided by information power.
23	Transcripts returned	Were transcripts returned to participants for comment and/or correction?	N.A.
24	Number of data coders	How many data coders coded the data?	9-10
25	Description of the coding tree	Did authors provide a description of the coding tree?	9-10. Figure 1
26	Derivation of themes	Were themes identified in advance or derived from the data?	9-10
27	Software	What software, if applicable, was used to manage the data?	9
28	Participant checking	Did participants provide feedback on the findings?	N.A.
29	Quotations presented	Were participant quotations presented to illustrate the themes?	10-17
30	Data and findings consistent	Was there consistency between the data presented and the findings?	10-17
31	Clarity of major themes	Were major themes clearly presented?	10-17
32	Clarity of minor themes	Is there a description of diverse cases or discussion of minor themes?	10-17
