# Supplementary material for: Beyond Self-Blame: A Qualitative Study of the Changing Experience of Parental Guilt Following the Death of a Child
Source: Behav Sci (Basel). 2026 Sep 28;16(10):1771. doi: 10.3390/bs16101771 (PMC13701316; doi:10.3390/bs16101771)
Supplement: Supplementary file 1 [file behavsci-16-01771-s001.zip › Table S1.pdf]

SUPPLEMENTARY MATERIALS.

Table S1. Participant Characteristics

Participant	Sex	Age of child at death	Time Since Child's Death	Cause of Child's Death
P1	Female	24 years	5 years 10 months	Traffic accident
P2	Female	8 years	1 year	Non-traffic accident
P3	Female	31 years	6 years	Oxygen deprivation
P4	Female	8 years	1 year 4 months	Traffic accident
P5	Female	15 years	2 years 4 months	Cerebral hemorrhage
P6	Male	20 years	1 year 7 months	Cancer
P7	Male	30 years	4 years 9 months	Tetraplegia
P8	Female	44 years	1 year 4 months	Suicide
P9	Female	19 years	3 years 8 months	Suicide
P10	Female	34 years	9 years	Cancer
P11	Female	20 years	1 year 3 months	Traffic accident
P12	Female	10 months	60 years	Heart disease
P13	Female	26 years	1 year 4 months	Traffic accident
P14	Female	20 years	6 years 1 month	Allergy
P15	Female	20 years	1 year 7 months	Cancer
P16	Male	20 years	1 year 3 months	Traffic accident
P17	Male	4 years	6 months	Drowning
P18	Female	18 years 10 months	2 years 6 months	Non-traffic accident
P19	Female	19 years	2 years	Cancer
P20	Male	19 years	5 years	Traffic accident
P21	Female	31 years	3 years	Traffic accident
P22	Female	19 years	5 years	Traffic accident
P23	Female	29 years	6 years 6 months	Traffic accident
P24	Female	33 years	8 months	Bacterial meningitis
P25	Female	17 years	13 years	Cancer
P26	Female	21 years	2 years	Traffic accident
P27	Female	16 years	7 years	Non-traffic accident
